# Biological and physicochemical properties of biosurfactants produced by *Lactobacillus jensenii* P_6A_ and *Lactobacillus gasseri* P_65_

**DOI:** 10.1186/s12934-017-0769-7

**Published:** 2017-09-19

**Authors:** I. M. C. Morais, A. L. Cordeiro, G. S. Teixeira, V. S. Domingues, R. M. D. Nardi, A. S. Monteiro, R. J. Alves, E. P. Siqueira, V. L. Santos

**Affiliations:** 1 Laboratório de Microbiologia Aplicada, Departamento de Microbiologia, Instituto de Ciências Biológicas, Universidade Federal de Minas Gerais, C.P. 486, Belo Horizonte, MG 31270-901 Brazil; 2 Laboratório de Microbiologia Aplicada, Universidade CEUMA, R. Josué Montello, 01, São Luís, MA 65075120 Brazil; 3 Departamento de Ciências Farmacêuticas, Faculdade de Farmácia, Universidade Federal de Minas Gerais, C.P. 486, Belo Horizonte, MG 31270-901 Brazil; 4 Laboratório de Química de Produtos Naturais, Centro de Pesquisas René Rachou, Fundação Oswaldo Cruz, Av. Augusto de Lima, 1715, Belo Horizonte, MG 30190-002 Brazil

**Keywords:** Biosurfactants, Antibiofilm, Antimicrobial, *Lactobacillus jensenii*, *Lactobacillus gasseri*

## Abstract

**Background:**

*Lactobacillus* species produce biosurfactants that can contribute to the bacteria’s ability to prevent microbial infections associated with urogenital and gastrointestinal tracts and the skin. Here, we described the biological and physicochemical properties of biosurfactants produced by *Lactobacillus jensenii* P_6A_ and *Lactobacillus gasseri* P_65_.

**Results:**

The biosurfactants produced by *L. jensenii* P_6A_ and *L. gasseri* P_65_ reduced the water surface tension from 72 to 43.2 mN m^−1^ and 42.5 mN m^−1^ as their concentration increased up to the critical micelle concentration (CMC) values of 7.1 and 8.58 mg mL^−1^, respectively. Maximum emulsifying activity was obtained at concentrations of 1 and 5 mg mL^−1^ for the P_6A_ and P_65_ strains, respectively. The Fourier transform infrared spectroscopy data revealed that the biomolecules consist of a mixture of carbohydrates, lipids and proteins. The gas chromatography-mass spectrum analysis of *L. jensenii* P_6A_ biosurfactant showed a major peak for 14-methypentadecanoic acid, which was the main fatty acid present in the biomolecule; conversely, eicosanoic acid dominated the biosurfactant produced by *L. gasseri* P_65_. Although both biosurfactants contain different percentages of the sugars galactose, glucose and ribose; rhamnose was only detected in the biomolecule produced by *L. jensenii* P_6A_. Emulsifying activities were stable after a 60-min incubation at 100 °C, at pH 2–10, and after the addition of potassium chloride and sodium bicarbonate, but not in the presence of sodium chloride. The biomolecules showed antimicrobial activity against clinical isolates of *Escherichia coli* and *Candida albicans*, with MIC values of 16 µg mL^−1^, and against *Staphylococcus saprophyticus*, *Enterobacter aerogenes* and *Klebsiella pneumoniae* at 128 µg mL^−1^. The biosurfactants also disrupted preformed biofilms of microorganisms at varying concentrations, being more efficient against *E. aerogenes* (64%) (P_6A_ biosurfactant), and *E. coli* (46.4%) and *S. saprophyticus* (39%) (P_65_ biosurfactant). Both strains of lactobacilli could also co-aggregate pathogens.

**Conclusions:**

This report presents the first characterization of biosurfactants produced by *L. jensenii* P_6A_ and *L. gasseri* P_65_. The antimicrobial properties and stability of these biomolecules indicate their potential use as alternative antimicrobial agents in the medical field for applications against pathogens that are responsible for infections in the gastrointestinal and urogenital tracts and the skin.

## Background

Microorganisms are able to produce diverse surface-active compounds (SACs) containing both hydrophilic and hydrophobic moieties that can interact with surfaces, lower surface and interfacial tensions, form micelles, and emulsify immiscible substances [1]. Microbial SACs can be distinguished by their size, such as low-molecular-weight biosurfactants and high-molecular-weight surface-active polymers. High-molecular-weight surface-active polymers can be amphiphilic or polyphilic [2]. The former possesses one hydrophobic region at one end of the molecule; examples include lipopolysaccharides, lipoteichoic acids and lipoglycans of bacterial cell walls. In contrast, the latter have hydrophobic groups distributed across the entire molecule that are identical to the hydrophobically modified, comb-type polymers; examples include emulsan and hydrophobic polysaccharides [2]. An additional criterion for categorizing microbial SACs is the chemical nature of the molecules. The major classes of molecules consist of various structures, such as glycolipids, lipopeptides, polysaccharides or protein complexes, phospholipids, fatty acids and neutral lipids [3]. These biomolecules can be transported to the extracellular medium or remain attached to the cell surface as particulate biosurfactants [4].

In recent years, interest in SACs have increased due to their possible applications in environmental protection, crude oil drilling and the food processing and pharmaceutical industries [5, 6]. Unlike chemical surfactants, which are primarily derived from petroleum, these molecules can be produced by a wide variety of microorganisms, including bacteria, yeasts and filamentous fungi [7–11]. Furthermore, biosurfactants have several advantages over chemical surfactants, including the following: low toxicity, a lower critical micelle concentration (CMC), higher intrinsic biodegradability, greater stability at temperature, pH, and salinity extremes, the possibility of being produced from renewable substrates, and greater ecological acceptability [12].

SAC-producing *Lactobacillus* species has been described and are predominately found among the urogenital and gastrointestinal tract microbiota of humans. SACs derived from lactic acid bacteria (LAB) contribute to the bacteria’s ability to prevent microbial infections associated with its ecosystems [13, 14]. Lactobacilli can prevent colonization of the urogenital tract by several pathogens, including yeasts of the genera *Candida albicans*, *C. tropicalis* and *C. krusei*, responsible for vulvovaginal candidiasis, anaerobic bacteria responsible for bacterial vaginosis (BV), such as *Gardnerella vaginalis*, *Mycoplasma hominis*, *Atopobium vaginae*, *Prevotella* spp., *Veillonella* spp. and *Mobiluncus* spp., the uropathogens *Escherichia coli*, *Proteus* spp., *Klebsiella* spp., *Serratia* spp. and sexually transmitted viruses [13–18].

Lactobacilli modulate the microbiota at these sites via different mechanisms, such as auto-aggregation, i.e., the ability to form multi-cellular aggregates that incorporate bacteria from the same species; lactic acid, hydrogen peroxide, bacteriocin, and SAC production; co-aggregation with pathogenic microorganisms (in which different bacterial species are incorporated); and adhesion to epithelial cells excluding pathogens [13, 17, 19]. This hypothesis of microbiota modulation has stimulated research on the isolation and characterization of novel SAC-producing lactobacilli, followed by investigations of the potential of these microorganisms to control pathogens.

Many studies have previously reported the antibacterial, antifungal and antiviral activities of SACs produced by lactobacilli [11, 20–22]. However, another valuable application of SACs is their use as anti-adhesive agents to prevent pathogen adhesion to the host epithelium and solid surfaces as biomedical instruments [21–26]. Thus, these biomolecules might constitute a new and effective method to prevent host colonization by pathogenic microorganisms and the consequent development of clinical disturbs.

The production yields of bacterial SACs are relatively high (2–10 g L^−1^). Additionally, SACs reduce the surface tension of water to values lower than 30 mN m^−1^. In contrast, biosurfactants produced by lactobacilli are less effective, only reducing the surface tension of water to values of approximately 36–40 mN m^−1^, and are produced at lower levels (20–100 mg L^−1^) [7, 20–22, 27, 28]. Furthermore, the chemical compositions of these biomolecules have not been well studied, with only a few biomolecules being partially characterized [10, 20–22, 29], and these characteristics can influence the biological activities of the SACs.

In general, human vaginal communities are dominated by one of the four more common *Lactobacillus* species, *L. gasseri, L. jensenii, L. crispatus,* and *L. iners* [30]. In this study, we characterized the antimicrobial activity of purified SACs from two *Lactobacillus* species (*L. jensenii* P_6A_ and *L. gasseri* P_65_) isolated from vaginal samples obtained from healthy women against clinical isolates of urogenital bacterial pathogens and the reference samples of the yeasts, *C. albicans, C. krusei* and *C. tropicalis.* These strains were previously characterized as able of antagonizing sixteen reference bacterial strains as demonstrated by in vitro assays [31]. However, the authors did not characterize the chemical nature of the active biomolecules produced by strains of *Lactobacillus* nor its effectiveness in controlling biofilms. Then, the physicochemical characterization of SACs was also performed, including the determination of the minimum surface tension, critical micelle concentration, stability at various pH values, temperatures and salt concentrations, as well as the evaluation of their chemical composition. It has been suggested that biofilm formation is an important virulence determinant in BV and other disorders of the genitourinary tract [32, 33]. Thus, the evaluation of the antibiofilm activities of biomolecules of these strains is important to validate their use as probiotic products to prevent urogenital infections. Besides this feature, the auto-aggregation activity and co-aggregation of these strains with pathogens were also studied.

## Methods

### Strains and culture conditions

In this study two strains of *Lactobacillus* (*L. jensenii* P_6A_ and *L. gasseri* P_65_) isolated from the vaginal fluids of healthy women were employed [31]. The *Lactobacillus* strains are non-H_2_O_2_-producing, that show antagonistic activity against strains of *Gardnerella vaginalis* isolated from healthy women and from women with BV, as demonstrated by in vitro assays. In this study, the strains were evaluated in terms of their production of biosurfactants with antimicrobial and anti-adhesive activities against uropathogens. The strains were stored at −80 °C in conventional synthetic de Man, Rogosa and Sharpe (MRS) broth (Difco, Detroit, MI, USA) with 15% (v/v) glycerol until further use [34]. Bacteria from a frozen stock were streaked on MRS agar plates and incubated overnight at the optimum growing temperature (37 °C) for further culturing. The agar plates were stored at 4 °C for no longer than 2 weeks. The following strains were used for the antimicrobial and anti-adhesive assays: urogenital tract clinical isolates of *E. coli*, *Klebsiella pneumoniae, Enterobacter aerogenes* and *Staphylococcus saprophyticus,* and reference strains of *C. albicans* ATCC 18804, *C. krusei* ATCC 20298, and *C. tropicalis* ATCC 750. All the bacterial strains were cultured in brain heart infusion (BHI) broth (Difco, Detroit, MI, USA) at 37 °C for 24 h, and the yeast were cultured on Sabouraud Dextrose agar (SD) (Oxoid, Basingstoke, UK) at 30 °C for 48 h.

### Biosurfactant production and isolation

For biosurfactant production, *L. jensenii* P_6A_ and *L. gasseri* P_65_ were cultured at 37 °C in 1 L Erlenmeyer flasks containing 600 mL of MRS broth (Difco, Detroit, MI, USA) on a rotatory shaker at 120 rpm. Six milliliters of an overnight culture were used for inoculations. After 72 h, the cells were harvested by centrifugation (10,000×*g* for 5 min at 10 °C), washed twice in demineralized water, and suspended in 100 mL of phosphate-buffered saline solution (PBS; 10 mM KH_2_PO_4_/K_2_HPO_4_ and 150 mM NaCl, pH adjusted to 7.0). Cell suspensions were incubated at room temperature for 2 h with gentle stirring to release the biosurfactant, as previously described [27, 28]. The cells were then removed by centrifugation, and the supernatant was dried in an oven at 70 °C. To confirm biosurfactant production, the emulsifying activity (E_24_), using toluene as the hydrophobic substrate, and surfactant activity were routinely measured. The biomolecules were extracted by acid precipitation according to the protocol described by Van Hoogmoed et al. [35]. Briefly, the extracts were suspended in PBS (pH 7.0) at a concentration of 10 mg mL^−1^, and the pH was adjusted to 2.0 with 1 M HCl. The acidified samples were incubated at 4 °C for 2 h, and the precipitates were collected by centrifugation (10,000×*g* for 15 min at 4 °C) and washed twice with acidic water (pH 2.0). The precipitates were dissolved in distilled water and adjusted to pH 7.0 using 1 M NaOH.

### Physicochemical properties

#### Surface–activity determination and critical micelle concentration (CMC)

The surface tension of the PBS extracts was measured using a KRUSS tensiometer (K10T model, Hamburg, Germany) with the plate method, and the relationship between the biosurfactant concentration and the surface tension was determined. To increase the accuracy of the surface tension measurements, the average value of triplicate determinations was calculated. All measurements were performed at room temperature (25 °C). The CMC was determined by plotting the surface tension as a function of the biosurfactant concentration and by locating the point of the intersection between the two lines that best fit through the pre- and post-CMC data. Concentrations ranging from 0.1 to 50 mg mL^−1^ were used in the assays.

#### Effects of the biosurfactant concentrations and the organic phase on the emulsifying activity

The extract was diluted in deionized water to concentrations ranging from 0.1 to 20 mg mL^−1^ to determine the effect of the biosurfactant concentration on emulsifying activity. In these emulsification assays, 1 mL of the solution at each concentration was added to screwcap tubes containing 1.5 mL of toluene, followed by homogenization using a vortex mixer at maximum speed for 2 min. After allowing the sample to stand for 24 h, emulsifying activity (E_24_) was determined using the method described by Cameron et al. [36]. The assays were performed in triplicate. The means were compared by Tukey’s test at 5% probability.

To evaluate the spectra of the emulsifying activity, E_24_ assays were performed using 5 mg mL^−1^ biosurfactant extracts and the following hydrophobic substrates: hexadecane (Sigma, St. Louis, MO), hexane (Sigma, St. Louis, MO), diesel oil (Petrobras, Brazil), gasoline (Petrobras, Brazil), kerosene (Petrobras, Brazil), olive oil (Food-Bunge), cottonseed oil (Food-Bunge), sunflower oil (Food-Bunge) and toluene (Sigma, St. Louis, MO).

### Characterization of biosurfactants

The protein concentrations of the biosurfactants were determined using Lowry’s method [37]. The total carbohydrate concentrations were quantitatively determined using a colorimetric method with glucose as the standard [38]. The method developed by Piretti et al. [39] was used to quantify lipids.

#### Fatty acid analysis

Fatty acids were analyzed by gas chromatography–mass spectrometry after conversion to their methyl esters derivatives. To determine the fatty acids composition, crude biosurfactants (2–5 mg) were hydrolyzed with aqueous 2 mol L^−1^ HCl at 100 °C for 2 h in a sealed tube. The free lipids were extracted using *n*-hexane dried and then methylated with a 14% boron fluoride-methanol reagent (Sigma-Aldrich, St. Louis, MO, USA) at a ratio of 1 mL of the reagent per 10 mg of lipids. The resulting sample was stored in 2 mL microtubes and incubated in a 95 °C water bath for 15 min [40]. Fatty acid methyl esters (FAMEs) were extracted three times with *n*-hexane and analyzed by gas chromatography and mass spectrometry (GC–MS) using a Shimadzu GC–MS model QP 5050 A (Shimadzu, Kyoto, Japan) equipped with a PTE-5-Supelco column (30 m × 0.25 mm ID, 0.25 µm film) and employing He as the carrier gas at 0.8 mL min^−1^, split of 20 and 50 kPa pressure. The column temperature was programmed to increase from 80 °C (1 min) to 180 °C at 20 °C min^−1^, increases of 3 °C min^−1^ until 240 °C, and a following 20 °C min^−1^ increase until 300 °C and then to be maintained at this temperature for 2 min. Electron impact spectra in positive ionization mode were acquired between *m*/*z* 50 and 500. The identification of the compounds was performed by means comparison of the retention time, mass fragmentation profiles and molecular ion between sample and standard of FAME (Supelco 37 component FAME MIX, Bellefonte, PA, USA). The results were recorded and processed using Class 3.02 software (Shimadzu) and expressed as the relative percentages of each FAME. The mass spectrum of each fatty acid methyl ester was matched with the National Institute of Standards and Technology *(*NIST) database.

#### Monosaccharide analysis

The carbohydrate composition of the biosurfactants was determined by analyses of their polyacetal derivatives using GC–MS. A lyophilized sample of biosurfactant (1 mg) was hydrolyzed with 150 µL 2 M trifluoroacetic acid (CF_3_COOH) in a sealed tube at 120 °C for 4 h. After evaporation, the residue was washed twice using methanol. The sample was then reduced with 1 M aqueous sodium borohydride (NaBH_4_, 100 µL) and acetylated with a mixture of potassium acetate (100 µg) and acetic anhydride (100 µL) at 100 °C for 2 h. Excess reagent was removed by evaporation and the sample was washed several times with ethanol. Alditol acetates were extracted with ethyl acetate and water (1:1, v:v) and analyzed using a Shimadzu GC–MS model QP 5050 A (Shimadzu, Kyoto, Japan) equipped with a PTE-5-Supelco column (30 m × 0.25 mm ID, 0.25 µm film) employing He as the carrier gas at 0.7 mL min^−1^, split of 10 and 40 kPa pressure. The column temperature was programmed to increase from 100 °C (1 min) to 200 °C at 4 °C min^−1^, followed by a 20 °C min^−1^ increase until 300 °C and then to be maintained at this temperature for 5 min. Electron impact spectra in positive ionization mode were acquired between *m*/*z* 40 and 400. The identity of the sugars was first confirmed by comparing with the retention time obtained from the individual monosaccharide standard by means of addition of sample in the mixture of standard (SUPELCO, Bellefonte, PA, USA) and were further identified through GC/MS coupled to the NIST database.

#### Fourier transmission infrared spectroscopy (FTIR)

The biosurfactants functional groups were further analyzed using FTIR [41]. Pellets for the infrared analysis were obtained by grinding a mixture of 1 mg EPS with 100 mg of potassium bromide. FTIR spectra were recorded in the region of 4000–650 cm^−1^ at a resolution of 4 cm^−1^ on a Spectrum-One FTIR spectrometer (Perkin Elmer, Shelton, CT, USA) using an attenuated total reflectance (ATR) system.

#### Stability assays

The stability of the biosurfactants under different physicochemical conditions was evaluated using a solution of 5 mg mL^−1^ crude biosurfactant. To examine the influence of pH on the emulsification index, the biomolecule was eluted in different buffer solutions with different pH values. The sample was then subjected to the emulsification assay (E_24_) using toluene as the organic layer. A 200 mM potassium chloride/hydrochloric acid buffer was used for measurements at pH 1 and 2; a 200 mM sodium acetate/acetic acid buffer for measurements at pH 3, 4 and 5; a 100 mM sodium phosphate buffer for measurements at pH 6, 7 and 8; and a 100 mM glycine/sodium hydroxide buffer for measurements at pH 9 and 10.

Furthermore, the heat stability of the crude biosurfactants was determined by incubating the biosurfactant solution (50 mg mL^−1^) in a 100 °C water bath for 60 min, and then cooling at room temperature. The emulsifying activity (E_24_) of each sample was determined as described above.

The effects of different sodium chloride (NaCl), potassium chloride (KCl) and sodium bicarbonate (NaHCO_3_) concentrations on the biosurfactant activity were evaluated by adding different concentrations of the salts (800, 1200 and 2000 µg mL^−1^) as the emulsion formed. The solutions were allowed to stand for 20 min, and then the emulsification indexes (E_24_) of the biosurfactants were measured. All assays were performed in triplicates.

### Antimicrobial activity assays

#### Culture media and inocula

Mueller–Hinton broth (Himedia, Maharashtra, India) was prepared in accordance with the CLSI document M7-A10 for minimal inhibitory concentration (MIC) bacterial assays [42]. The inocula of all bacteria at final concentration of 10 × 10^5^ CFU mL^−1^ were prepared using the spectrophotometric method. *Candida* cultures were freshly grown at 35 °C. For the susceptibility tests, inoculum suspensions were prepared at final concentrations of 1–5 × 10^3^ cells mL^−1^ using the spectrophotometric method, in accordance with CLSI document M27-A3 [43].

#### Susceptibility tests

The broth microdilution method was performed in accordance with the guidelines of the CLSI document M7-A6 for bacteria and M27-A3 for yeast using flat-bottom 96-well microplates (Corning, NY, USA). Stock solutions of biosurfactants were prepared in water at a concentration of 1024 µg mL^−1^. The compounds were diluted 1:2 in Mueller–Hinton broth to obtain a concentration two-fold greater than the maximum concentration in the analysis. Serial dilutions were prepared from this solution using the medium as a diluent. The compounds were tested at concentrations ranging from 256 to 4 µg mL^−1^. For tests using yeast, the stock solutions were prepared in RPMI 1640 medium (Sigma-Aldrich, St. Louis, MO, USA). Media without the extract and the solvent were used as growth and sterility controls. Chloramphenicol (Sigma-Aldrich; 0.78–100 µg mL^−1^) was used as a positive antibacterial control, and amphotericin B (Sigma-Aldrich; 0.03–15 µg mL^−1^) was used as a positive antifungal control. After plate assembly, 100 µL of each bacterial and yeast strain was inoculated per well in order to obtain 5 × 10^5^ CFU mL^−1^ (or 5 × 10^4^ CFU per well) and 0.5–2.5 × 10^3^ CFU mL^−1^ (or 0.5–2.5 × 10^2^ CFU per well), respectively. Then, the plates were incubated at 37 °C for 24 h for bacteria and 48 h for *Candida* species. All tests were performed in triplicate in at least two independent experiments. The MIC was defined as the lowest concentration of biosurfactants that completely inhibited the visible growth of test microorganisms.

### Biosurfactant-mediated disruption of pre-formed biofilms

The antibiofilm activity of the biosurfactants against several microbial strains was determined using the procedure described by Heinemann et al. [44]. Bacterial isolates were grown in BHI for 24 h at 37 °C and yeast were grown in RPMI for 48 h at 30 °C. After incubation, the cells were centrifuged at 7200×*g* for 15 min, washed twice with PBS and used to prepare an inoculum at a density equivalent to 0.5 on the McFarland scale.

180 µL of BHI broth containing 1% glucose (bacteria) or RPMI (yeast) and 20 µL of the standardized inocula were added to each well of untreated 96-well polystyrene plates (Corning, NY, USA). The plates were then incubated for 24 h at 37 °C for bacteria and at 30 °C for yeasts. After incubation, unattached cells were removed by washing the wells, and biosurfactant at concentrations ranging from 180 to 22.5 mL L^−1^ were then added. The plates were further incubated under the same conditions.

The assay was performed with four replicates of the control (medium without extract/biosurfactant) and four replicates of each concentration of the extract/biosurfactant studied. Non-adherent cells were removed using a multichannel pipette and the wells were washed three times with PBS. Cells that adhered to the bottoms of the wells (biofilm) were fixed with 300 µL of 99% methanol and stained for 5 min with a solution of 1% crystal violet. The excess stain was removed by placing the plate under running tap water. The plates were then air-dried and the dye that bound to the adherent cells was solubilized with 200 µL 95% ethanol. The solutions were transferred to another polystyrene plate and the absorbance was measured at 450 nm using a Multiskan MMC/340 microplate reader (Thermo Scientific GO, Waltham, MA, USA). The percentage of biofilm disruption was assessed by comparing the absorbance readings of the wells treated with the extract/biosurfactant and the control wells (not treated with the extract/biosurfactant).

### Auto-aggregation and co-aggregation assays and cell surface hydrophobicity

Auto-aggregation assays were performed using the method reported by Vandevoorde et al. [45]. *L. jensenii* P_6A_ and *L. gasseri* P_65_ were grown in flasks containing 100 mL MRS broth for 48 h at 37 °C and 120 rpm. The cells were harvested by centrifugation (10,000×*g* for 10 min at 10 °C), washed twice in demineralized water, and re-suspended in PBS (pH 7) at an OD_600_ of 0.6 ± 0.5 (approximately 10^8^ CFU mL^−1^). The OD was measured using a spectrophotometer (Shimadzu CPS 240A) at regular intervals over a 4 h period, without disturbing the microbial suspension, and the sedimentation kinetics were obtained. The auto-aggregation coefficient (AC) was calculated at different times using the method reported by Kos et al. [46] as follows:

$$ACt = \frac{ODi - ODt}{ODt} \times 100$$

where ODt is the optical density at 600 nm of the microbial suspension at *t* time (0.5, 1, 2, 3 or 4 h), and ODi is the initial optical density.

The co-aggregation assay was performed using the same method as the auto-aggregation assay, the same pathogen isolates and two isolates of the LAB *L. jensenii* P_6A_ and *L. gasseri* P_65_. Equal volumes (2 mL) of each *Lactobacillus* suspension were added to the following pathogens: *E. coli, S. saprophyticus, E. aerogenes, K. pneumoniae, C. albicans, C. krusei, and C. tropicalis.* Then, the samples were vortexed for 15 s. Control tubes containing 4 mL of each bacterial suspension were prepared simultaneously. The OD of the suspensions was measured after the initial preparation and 4 h after incubation at 25 °C. The co-aggregation percentage was calculated using the equation reported by Handley et al. [47]:

$$Co\text{-}aggregation\,(\%) = \frac{{\left[ {\frac{{\left( {\text{ODx + ODy}} \right)}}{2} - {\text{OD}}({\text{x}} + {\text{y}})} \right]}}{{\frac{{\left[ {\text{ODx + ODy}} \right]}}{2}}} \times 100$$

where x and y represent OD measurements of tubes containing either the lactobacilli or the pathogen suspensions, respectively, and (x + y) represents OD measurements of tubes containing a mixture of the pathogen and *Lactobacillus* suspensions.

The two strains were treated with LiCl (5 M) and incubated for 30 min at room temperature to remove the S layer (cell surface proteins) and to evaluate the influence of the S layer in auto-aggregation and co-aggregation capacities. Auto- and co-aggregation assays were performed and the results were compared to the assays conducted with cells containing the S layer.

## Results and discussion

### Production and tensoactive properties of the SACs

The production of biosurfactants by *L. jensenii* P_6A_ and *L. gasseri* P_65_ during growth in MRS broth was monitored by verifying the emulsifying activity using toluene as organic phase and by measuring the surfactant activity of supernatant. The emulsifying and surfactant activities at 72 h of incubation corresponded to 63.75% and 56 mN m^−1^ for *L. jensenii* P_6A_ and 70% and 46 mN m^−1^ for *L. gasseri* P_65_. The production corresponded to 0.27 g L^−1^ for *L. jensenii* P_6A_ and 0.42 g L^−1^ for *L. gasseri* P_65_. This low production pattern has already been described for other LAB, with values ranging from 0.02 to 0.1 g L^−1^, whereas for genera as *Pseudomonas* and *Bacillus*, the yield varies from 2 to 15 g L^−1^ [20, 22, 27, 28, 48].

**Fig. 1:**
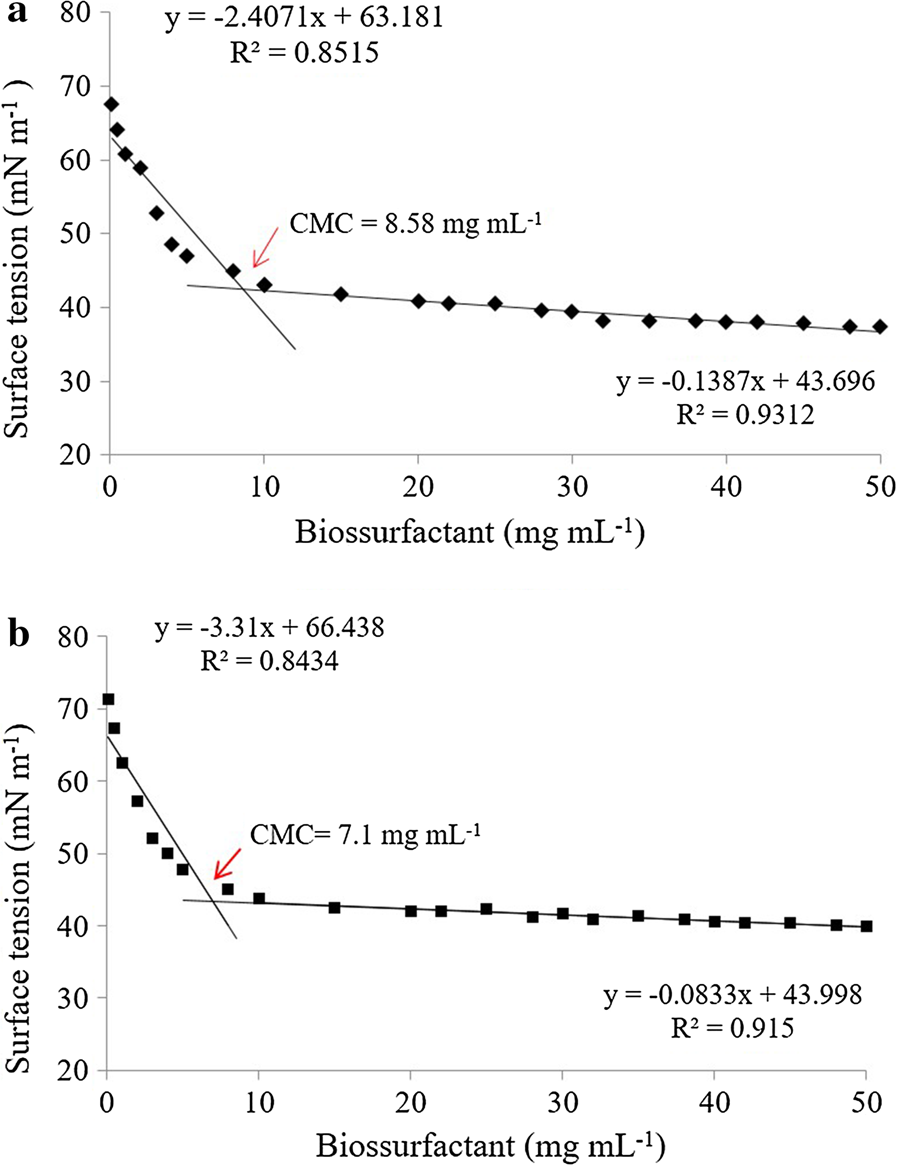
Effects of different concentrations of biosurfactants on the surface tension of water at room temperature (25 °C). **a** Surface tension (mN m^−1^) of the biosurfactants produced by *L. jensenii* P_6A_ and **b** *L. gasseri* P_65_. The CMC was determined from the intersection between the regression lines that better described the two parts of the curve, below and above the CMC (arrow). The results represent the average of two independent measurements

In the assays performed to establish the CMC of the crude biosurfactants isolated from *L. jensenii* P_6A_ and *L. gasseri* P_65_, a progressive decrease in surface tension was observed as the biosurfactant concentration increased. The CMC values of the biosurfactants from P_6A_ and P_65_ were calculated as 7.1 and 8.58 mg mL^−1^, respectively, as shown in Fig. 1. There are different mathematical methods based on parametric and nonparametric estimation of the regression function to CMC definition and of other features in different application fields with good results [49–51]. In this study, we used the method of simple linear regression. It was assumed that the regression function corresponds to a straight line, hence called regression line, and the estimation of this straight line is reduced to the estimation of two of its parameters, slope and intercept. The advantage of these parametric methods is that when the functional model assumed for X (CMC concentration in this case) is adequate, the estimation is reduced to a few parameters, and therefore, it is extremely efficient. For CMC determination, two lines were estimated and assumed that the intersection point of these lines indicates the precise CMC concentration and thus an indication for the biosurfactant concentration with the highest capacity for surface tension reduction. At the points corresponding to CMC, the biomolecules from *L. jensenii* P_6A_ and *L. gasseri* P_65_ reduced the water surface tension from 72 mN m^−1^ to approximately 43.2 and 42.5 mN m^−1^, respectively. The ability of a biosurfactant to reduce surface and interfacial tensions determines its functionality and effectiveness. For example, a good surfactant reduces the surface tension of water from 73.20 to 35.0 mN m^−1^ [52]. The values observed for biosurfactants from P_6A_ and P_65_ are in the range of those observed for sodium dodecylsulfate (SDS) and biosurfactants isolated from different lactobacilli strains and other lactic acid bacteria (LAB) [20, 22, 27, 28, 52].

**Fig. 2:**
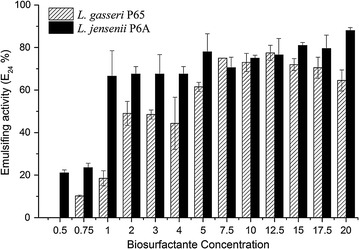
Effects of the concentration of biosurfactants produced by *L. jensenii* P_6A_ and *L. gasseri* P_65_ on emulsifying activity, expressed as the emulsification index (E_24_), using toluene as the organic phase

Relationships between the concentrations of the biosurfactants produced by *L. gasseri* P_65_ and *L. jensenii* P_6A_ and the emulsifying activity, expressed as the emulsification index (E_24_), were evaluated using toluene as organic phase (Fig. 2). In general, E_24_ values increased as the concentration of the biosurfactant increased to 20 mg mL^−1^. For the biosurfactant produced by P_6A_, E_24_ values ranged from 21% in tests containing 0.5 mg mL^−1^ to 88.7% in tests with 20 mg mL^−1^ biosurfactant. No emulsifying activity was observed in tests using concentrations lower than 0.5 mg mL^−1^, and the differences in values between 1 and 17.5 mg mL^−1^ were not significantly different (*p* > 0.05). For the biosurfactant produced by *L. gasseri* P_65_, the emulsification index ranged from 10% in tests with 0.75 mg mL^−1^ biosurfactant to 77% in tests with 12.5 mg mL^−1^ biosurfactant. At concentrations higher than 5 mg mL^−1^, there was no significant difference in emulsifying activity (*p* > 0.05) according to Tukey’s test.

**Fig. 3:**
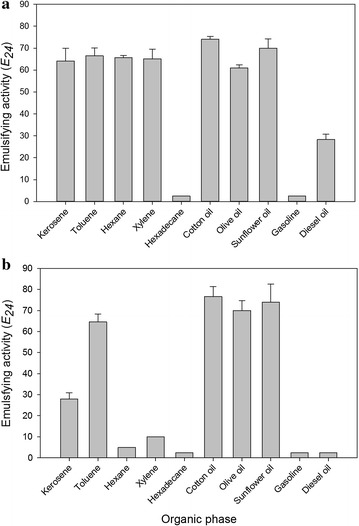
Emulsifying activities of 5 mg mL^−1^ biosurfactants produced by *L. jensenii* P_6A_ (**a**) and *L. gasseri* P_65_ (**b**) on the aqueous phase using different hydrophobic substrates, expressed as the emulsification index (E_24_)

The biosurfactants produced by *L. jensenii* P_6A_ and *L. gasseri* P_65_ presented different emulsification activities according to the different evaluated hydrophobic substrates. The biomolecule produced by *L. jensenii* P_6A_ emulsified kerosene, toluene, hexane and xylene organic solvents at values greater than 62% and diesel oil at 28.3%, but showed low levels of emulsification of gasoline and hexadecane (Fig. 3a). E_24_ values ranged from 61 to 70% for vegetable oils (cotton, olive, and sunflower oils). For the biosurfactant produced by *L. gasseri* P_65_ (Fig. 3b), high E_24_ values were observed in assays with vegetable oils (cotton, olive and sunflower oils), whereas low values were observed for gasoline, diesel oil, hexane, xylene and hexadecane. The values for kerosene and toluene were 28.0 and 64.6%, respectively.

The chemical structure of both the biosurfactants and the emulsions organic phases may explain the variations observed in the emulsifying activity saturation point and in the profile of the emulsified compounds. The different results observed for *L. jensenii* P_6A_ biosurfactant with diesel oil and gasoline can be explained by the fact that these substrates consist of a complex mixture of hydrocarbons, with the predominance of hydrocarbons of shorter carbon chains in gasoline and longer in diesel. Vegetable oils have a different hydrocarbon composition, mainly consisting of triglycerides. In general, the indexes found in our study were greater than the values reported for biosurfactants produced by other LAB. Emulsifying indexes between 40 and 49% with gasoline, kerosene and octane were observed for the biosurfactant produced by *L. pentosus* CECT4023 [53, 54]. In addition, the biosurfactant produced by *L. plantarum* CFR2194 exhibited emulsifying indexes between 13.6 and 38.2% for a variety of water-immiscible substrates [55]. The biomolecules from LAB strains (L26, L35 and L61) emulsified kerosene, sunflower oil, and olive oil with indexes varying from 8.22 to 26.5%, and the emulsions formed with the edible oils were more stable than the emulsions formed with kerosene [56]. The lipopeptide from *Bacillus subtilis* K1 isolated from aerial roots of banyan also showed good emulsification rates for olive oil [57].

### Characterization of the biosurfactant

**Fig. 4:**
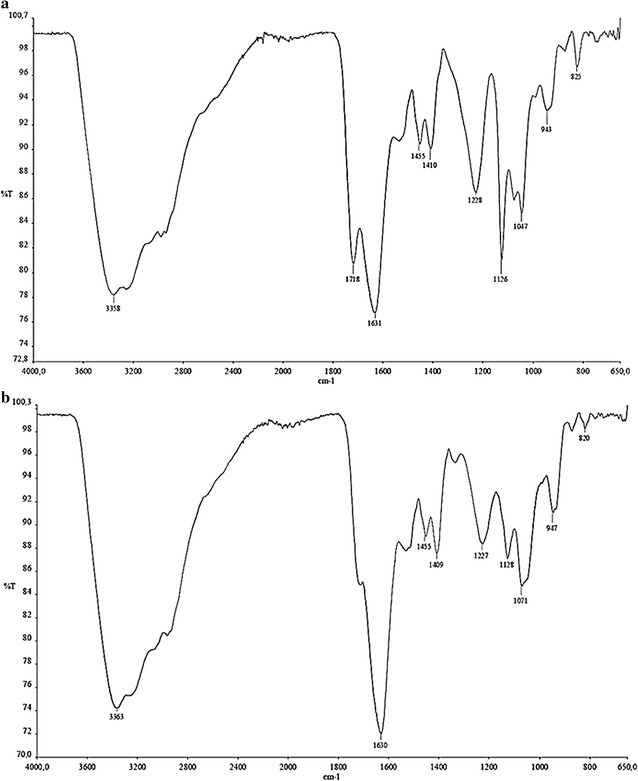
FTIR spectra of the biosurfactants produced by *L. jensenii* P_6A_ (**a**) and *L. gasseri* P_65_ (**b**)

The carbohydrate, protein and lipid concentrations of the *L. jensenii* P_6A_ and *L. gasseri* P_65_ biosurfactant extracts were determined. The carbohydrate concentrations ranged from 51.49 to 38.61% and the protein and lipid concentrations ranged from 15.17 to 9.81% and 29.45 to 49.53%, respectively. These results show diverse biosurfactant structures, which are confirmed by FTIR analysis (Fig. 4). FTIR is widely used to characterize the functional groups of organic compounds based on the characteristic infrared absorption bands of specific chemical groups [58]. The presence of a broad band at 3500–3200 cm^−1^ in the spectra of the biosurfactants produced by *L. gasseri* P_65_ and *L. jensenii* P_6A_ indicates the presence of OH groups (and, possibly, NH groups) of glycoproteins (Fig. 4; Table 1). Another band observed at approximately 1650 cm^−1^ corresponds to C=O stretching of peptide bonds. The absorption band observed in the region near 1720 cm^−1^, which was partly superimposed on the band at 1650 cm ^−1^, may be attributed to the C=O stretching of lipid esters. The absorption bands around 1230 and ~1100 cm^−1^ can be attributed to ester asymmetric and symmetric C–O–C stretching, respectively. Intense bands were also observed at 1100–1000 cm^−1^, indicating the presence of C–O sugar linkages.

**Table 1:** Correlation between FTIR spectra and functional groups detected in biosurfactants produced by *L. jensenii* P_6A_ and *L. gasseri* P_65_

Absorbance range (cm^−1^)	Functional groups detected
Below 1000	OH deformation vibrations/CN
1000–1300	C–O sugar stretching
1400–1460	C–H vibrations of groups CH_2_ e CH_3_
1520	Groups N–H in proteins
1725–1675	C=O stretching of carbonyl group
3200–3600	OH and NH stretching

Although little is known about the chemical structures of the biosurfactants produced by lactobacilli, some researchers have reported an initial characterization [53]. Indeed, it was found that *L. pentosus* biosurfactants are composed of 44.7 ± 1.5% soluble protein and 13.4 ± 2.9% total sugars; those obtained from *L. fermentum* B54 are rich in proteins, with few polysaccharides and phosphate groups [21]. These results are similar to those described for high molecular weight biosurfactants produced by bacteria and yeast, which are characterized by the presence of primarily 16- and 18-carbon fatty acids.

The biosurfactants produced by *L. gasseri* P_65_ and *L. jensenii* P_6A_ showed a different profile of fatty acids in the lipid portion, presenting only a fatty acid in common, the 14-methylpentadecanoic acid (16 carbon fatty acid) (Table 2). This fatty acid predominated on the biosurfactant produced by *L. jensenii* P_6A_, constituting 69% of the lipid fraction, while eicosanoic acid (20 carbon fatty acid) predominated in the biomolecule produced by *L. gasseri* P_65_, corresponding to 47.43%. Although the two biosurfactants contained the same sugars, the percentages varied. In addition, rhamnose was not detected in the biosurfactant produced by *L. gasseri* P_65._ These differences can explain the emulsified compounds profile, the E24 and CMC values and the stability profiles at different temperatures, pH and in the presence of different salts ions.

Several studies have reported similar structures for LAB. *Lactobacillus helveticus* produce a glycolipid-type biosurfactant closely resembling xylolipids [59]. The biosurfactants produced by *Lactococcus lactis* 53 are composed of glycoproteins with glucose, rhamnose, fucose and mannose [28]. The biosurfactant from *L. fermentum* B54 are also composed of a large amount of proteins, but fewer polysaccharides and phosphate groups [21]. The biosurfactants spectra produced by *L. lactis, L. paracasei* and *L. pentosus* showed bands at approximately 3200–3500 cm^−1^, characteristic of glycoprotein stretching [7, 29, 53]. Bands at approximately 1675 and 1725 cm^−1^, corresponding to C=O (carbonyl groups) and NH (peptides), respectively, have been observed in *L. pentosus* biosurfactant, whereas bands at 2900 and 1000–1200 cm^−1^ [53] indicate the presence of glycoproteins. The biosurfactants produced by *L. rhamnosus* 1825 and *L. fermenti* 126 CCM have also been evaluated, with bands at approximately 3285, 1635 and 1549 cm^−1^, typical of NH bonds and CO–N bonds in proteins found for the latter. Similar spectra were observed for *L. rhamnosus*. The bands at approximately 2964, 2929 and 1458 cm ^−1^ and 2961, 2936 and 1453 cm^−1^ for the biosurfactants produced by *L. fermenti* and *L. rhamnosus* CCM1825 126, respectively, correspond to the CH bonds of aliphatic groups; while peaks at 1200–1000 cm^−1^ confirm the presence of polysaccharide fractions [60]. When comparing infrared spectroscopic data for the compounds produced by *L. gasseri* P_65_ and *L. jensenii* P_6A_ with data reported for other biosurfactants produced by LAB, it can be concluded that the compounds reported in the present study are related to those produced by *L. lactis* and *L. paracasei*. These observations suggest that these substances have a complex structure composed of glycolipoproteins.

**Table 2:** Fatty acid and monosaccharide compositions of biosurfactants produced by *L. jensenii* P_6A_ and *L. gasseri* P_65_

Chemical composition	*L. jensenii* P_6A_	*L. gasseri* P_65_
	Concentration (%)	
Fatty acid		
9-Dodecenoic acid	31.0	–
12-Methyltetradecanoic acid	–	9.88
14-Methypentadecanoic acid	69.0	9.70
(*Z*)-9-Octadecenoic acid	–	16.91
Octadecanoic acid	–	16.08
Eicosanoic acid	–	47.43
Monosaccharides		
Galactose	38.12	25.50
Glucose	47.99	40.70
Rhamnose	10.44	–
Ribose	3.46	33.80

### Stability of the biosurfactants

The applicability of biosurfactants in several fields depends on their stability at different temperatures, pH values and salt concentrations. We observed stability of the biosurfactants produced by *L. jensenii* P_6A_ and *L. gasseri* P_65_ after 60-min incubation at 100 °C, with no loss of activity (data not shown). This profile is similar to previously reported results. Desai and Banat [4] found that heat treatment (autoclaving at 120 °C for 15 min) of *Bacillus* sp. biosurfactants did not cause appreciable changes in their surface and emulsifying activities, and an additional biosurfactant isolated from *L. paracasei* showed unaltered surfactant activity after 120 h of incubation at 60 °C [29].

The emulsions formed also remained relatively stable after standing for 24 h at pH values ranging from pH 2 to 10, maintaining values of approximately 66.34% (±1.12) (data not shown). The biomolecules are relatively more stable to pH variations than other biomolecules described for LAB. Gudiña et al. [29] observed precipitation of some of the biosurfactant components produced by *L. paracasei* at pH values lower than 6, which may have contributed to the alterations in the surface activities.

The addition of potassium chloride and sodium bicarbonate to the emulsions formed from toluene and the biosurfactants produced by *L. jensenii* P_6A_ and *L. gasseri* P_65_ did not affect the emulsions. Indeed, the emulsifying indexes of *L. jensenii* P_6A_ and *L. gasseri* P_65_ were maintained between 62 and 65.35% and between 58.43 and 65.54%, respectively, even when the salt concentration exceeded the limits of saturation (data not shown). However, when NaCl was added to the emulsions, there was an approximately 30% decrease in the height of the emulsified layer for *L. jensenii* P_6A_ biosurfactant and an approximately 31% decrease for *L. gasseri* P_65_ biosurfactant. This profile is already known; the emulsifying activity of commercial surfactants tends to decrease with NaCl increasing, falling at concentration about 10–12% [61].

### Antimicrobial activity

The evaluation of antimicrobial effects of biosurfactants of both *L. jensenii* P_6A_ and *L. gasseri* P_65_ showed similar MIC values corresponding to 16 µg mL^−1^ for *E. coli,* and 128 µg mL^−1^ for *K. pneumoniae, E. aerogenes* and *S. saprophyticus* (Table 3). Furthermore, the extracts at a concentration of 16 µg mL^−1^ completely inhibited *C. albicans* growth, but were not active against the other species of *Candida* (*C. krusei* and *C. tropicalis*) at the highest concentration tested (256 µg mL^−1^). Although there are few reports regarding the antimicrobial activity of biosurfactants isolated from lactobacilli, some have been reported to exhibit activity against various microorganisms. For example, biosurfactants isolated from *L. jensenii* and *L. rhamnosus* completely inhibited the growth of *Acinetobacter baumannii*, *E. coli* and *S. aureus* at 25–50 mg mL^−1^ [62]. But the authors did not chemically characterize the biosurfactants. In another study, biosurfactants produced by *Lactobacillus* strains inhibited the growth of *Pseudomonas* spp. isolated from fresh beef, with minimal inhibitory concentrations (MIC) ranging from 25 to 50 mg mL^−1^ [63]. The biosurfactants studied here exhibited MIC values that support more studies objecting its use in antibacterial therapies or probiotics.

**Table 3:** Antimicrobial activity of biosurfactants produced by *L. jensenii* P_6A_ and *L. gasseri* P_65_ on uropathogens

Strains	MIC of biosurfactants (µg mL^−1^)	
	*L. jensenii* P_6A_	*L. gasseri* P_65_
*Escherichia coli*	16	16
*Klebsiella pneumoniae*	128	128
*Enterobacter aerogenes*	128	128
*Staphylococcus saprophyticus*	128	128
*Candida albicans*	16	16
*Candida krusei*	n	n
*Candida tropicalis*	n	n

*n* the biosurfactants not inhibited the growth of yeasts at the highest concentration tested (256 µg mL^−1^)

### Disruption of pre-formed biofilms

**Fig. 5:**
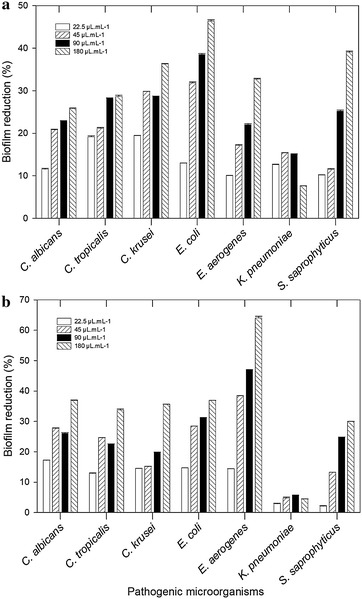
Percentage of disruption of biofilms produced by pathogenic microorganisms on the surface of polystyrene plates in the presence of different concentrations of biosurfactants produced by *L. gasseri* P_65_ (**a**) and *L. jensenii* P_6A_ (**b**). The test was conducted on four replicates after 24 h of incubation

The *Lactobacillus* biosurfactants disrupted the biofilms of all tested microorganisms at different levels. The greatest percentages of biofilm disruption were obtained in tests with 180 µL mL^−1^ of *L. jensenii* P_6A_ biosurfactant and *E. aerogenes* (64%) (Fig. 5a). For the other microorganisms, the disruption did not exceed 36% at the same concentration. The *L. gasseri* P_65_ biosurfactant decreased the formation of *E. coli* (46.4%) and *S. saprophyticus* (39%) biofilms in a high degree, but the values did not exceed 33% for the other microorganisms (Fig. 5b). Biosurfactants can adsorb to surfaces, forming a film at the interfaces by orienting polar and nonpolar groups according to the hydrophilicity/hydrophobicity of the surface. This interaction between biosurfactants and a substratum surface alters the surface hydrophobicity, thereby interfering with microbial adhesion and desorption processes [2, 28].

Diverse studies have highlighted the importance of biofilm formation as a virulence factor for uropathogens such as *G. vaginalis* and *E. coli* [32, 33]. The in vitro model of adherence and biofilm formation used in this study is limited by the fact that the use of polystyrene plates does not mimic the in vivo conditions in epithelial cells. However, despite their limitations, in vitro models can be very informative, and they are crucial for obtaining further understanding of activities of anti-biofilm compounds. The results showing the anti-biofilm activities of biomolecules produced by lactobacilli validate and/or explain their activities in vivo, for example, following intravaginal administration in a pharmaceutical form for the prevention and treatment of recurrent infections of the genital tract.

### Auto-aggregation and co-aggregation of *L. jensenii* P_6A_ and *L. gasseri* P_65_

Auto-aggregation of *L. jensenii* P_6A_ and *L. gasseri* P_65_ and their co-aggregation with pathogens were evaluated. The sedimentation rate of the *Lactobacillus* strains was measured over a 4-h period using washed cells resuspended in PBS (pH 7.0). Low sedimentation levels after 4 h were obtained for both strains, with values corresponding to 7.38 and 10.74% for *L. jensenii* P_6A_ and *L. gasseri* P_65_, respectively (data not shown). *L. jensenii* P_6A_ exhibited greater aggregation with *E. coli* (27.4%) and *C. albicans* (25.9%) in co-aggregation tests with pathogenic microorganisms; for *L. gasseri* P_65_, a higher co-aggregation score was found for *C. tropicalis* (11.9%) (Table 4). In general, the activities were higher in the Gram-negative bacteria assays (*E. coli*, *E. aerogenes* and *K. pneumoniae*) when compared to the Gram-positive (*S. saprophyticus*) ones.

**Table 4:** Co-aggregation activities of *L. jensenii* P_6A_ and *L. gasseri* P_65_ after 4 h of incubation in PBS

Pathogens	% agreggation					**% agreggation after S layer removal**				
	Oh	1h	2h	3h	4h	Oh	1h	2h	3h	4h
*Lactobacillus jensenii* P6A										
*Escherichia coli*	3.4 ± 0.7	26.8 ± 0.2	27.5 ± 0.5	27.8 ± 0.0	27.4 ± 0.1	1.4 ± 0.5	2.8 ± 0.6	1.5 ± 0.2	1.2 ± 0.0	1.4 ± 0.4
*Staphylococcus saprophytics*	5.9 ± 0.6	7.8 ± 0.2	8.6 ± 0.3	7.9 ± 0.0	7.4 ± 0.3	0.7 ± 0.2	3.5 ± 0.0	2.8 ± 0.0	8.9 ± 0.0	2.4 ± 0.2
*Enterobacter aerogenes*	6.6 ± 0.7	9.9 ± 4.4	9.7 ± 2.8	10.6 ± 5.2	10.3 ± 4.8	2.8 ± 0.1	1.5 ± 0.4	0.1 ± 0.8	2.7 ± 5.2	3.3 ± 0.4
*Klebsiella pneumoniae*	17.3 ± 0.6	10.2 ± 0.2	7.3 ± 0.4	10.4 ± 0.1	7.2 ± 0.4	14.8 ± 0.2	8.7 ± 0.2	6.2 ± 0.5	11.1 ± 0.2	5.2 ± 0.1
*Candida albicans*	10.3 ± 0.6	13.5 ± 0.2	18.5 ± 0.3	25.6 ± 0.1	25.9 ± 0.4	8.7 ± 0.3	10.5 ± 0.1	15.2 ± 0.2	17.1 ± 0.0	15.2 ± 0.6
*Candida krusei*	6.9 ± 0.7	16.7 ± 0.9	1.5 ± 0.5	4.5 ± 0.1	6.9 ± 0.5	6.03 ± 0.4	10.1 ± 0.3	12.2 ± 0.4	13.6 ± 0.6	9.4 ± 0.7
*Candida tropicalis*	7.6 ± 0.7	20.2 ± 0.2	13.7 ± 0.6	8.8 ± 0.2	3.1 ± 0.6	4.9 ± 0.0	10.3 ± 0.0	11.8 ± 0.0	6.4 ± 0.1	0.1 ± 0.5
*Lactobacillus gasseri* P65										
*Escherichia coli*	5.5 ± 2.8	3.3 ± 2.6	3.1 ± 3.6	2.6 ± 3.4	4.5 ± 11	0.5 ± 0.2	1.3 ± 0.2	2.1 ± 0.8	1.7 ± 0.5	2.5 ± 0.1
*Staphylococcus saprophytics*	6.5 ± 2.6	4.6 ± 2.4	6.0 ± 3.2	7.8 ± 3.0	6.6 ± 5.2	1.2 ± 0.6	13.8 ± 0.4	0.2 ± 0.3	6.4 ± 0.6	4.8 ± 0.5
*Enterobacter aerogenes*	2.6 ± 2.9	5.9 ± 2.7	5.9 ± 3.6	7.8 ± 3.6	9.7 ± 6.2	3.6 ± 0.2	2.5 ± 2.7	0.5 ± 0.6	3.5 ± 0.7	6.8 ± 0.6
*Klebsiella pneumoniae*	4.2 ± 2.9	3.6 ± 2.7	3.4 ± 3.7	2.6 ± 3.1	4.4 ± 1.5	5.8 ± 0.8	6.3 ± 0.2	9.9 ± 0.7	3.4 ± 0.3	2.6 ± 0.1
*Candida albicans*	8.9 ± 2.7	2.3 ± 0.9	8.4 ± 5.6	10.7 ± 5.9	7.5 ± 4.1	5.6 ± 0.6	1.9 ± 0.6	6.0 ± 0.6	7.2 ± 0.5	4.7 ± 0.6
*Candida krusei*	2.2 ± 1.3	6.2 ± 2.8	3.1 ± 4.2	6.1 ± 3.9	6.2 ± 0.4	2.5 ± 0.1	6.6 ± 0.7	1.8 ± 0.2	4.6 ± 0.6	2.1 ± 0.6
*Candida tropicalis*	9.0 ± 2.6	10.9 ± 2.4	25.4 ± 6.9	9.6 ± 5.9	11.9 ± 1.0	8.5 ± 2.6	9.9 ± 0.6	15.2 ± 0.7	7.8 ± 0.4	10.1 ± 0.5

The average results of two separate experiments are shown

Auto-aggregation of lactobacilli appears to be necessary for adhesion to epithelial cells and mucosal surfaces, while co-aggregation with pathogens has been considered a strategy to exclude pathogenic bacteria from their hosts [46, 64]. The very close proximity of bacteria on the aggregate permit that antimicrobial substances released by lactobacilli can directly inhibit the pathogens. The lactobacilli in our study did not show a substantial self-aggregation ability, which differs from a previous study reporting auto-aggregation indexes of 51% for *Lactobacillus paracasei* strains, 45% for *L. acidophilus* M92 and 58% for *Lactobacillus kefir* 2345 [29, 65]. The co-aggregation scores with pathogens also were lower as compared to other studies. As an example, a strain of *L. plantarum* showed a co-aggregation score of 41.5% with enterohemorrhagic *E. coli* (EHEC), 40.5% with *Salmonella enterica* serotype Typhimurium, and 37.4% with *Listeria monocytogenes* [66]. The co-aggregation activity of lactobacilli can be variable and appears to be dependent on the strain used in the tests, as observed for *L. delbrueckii* L10. This strain could co-aggregate with *S. aureus* 1351 and *C. albicans* ATCC 70014, at percentages of 48.88 and 59.37%, respectively [67]. In contrast, Pan et al. [68] reported co-aggregation capacities of *L. acidophilus* and *L. delbrueckii* with *Clostridium butirycum* of only 5.76% ± 6.32 and 1.2 ± 1.6, respectively.

The auto-aggregation rates of the *Lactobacillus* sp. strains decreased after removing the S layer, showing percentages of up to 5.0 and 6.8% for *L. jensenii* P_6A_ and *L. gasseri* P_65_, respectively (data not shown). Similarly, a decrease in co-aggregation capacity with the pathogens was found. The greatest reductions after removal of the S layer were between *L. jensenii* P_6A_ and *E. coli*, with values ranging from 27.4 to 1.4% (Table 4). Some authors have reported the importance of this protein layer for adhesion of *Lactobacillus* spp. For example, Kos et al. [46] showed that the removal of the protein layer decreased the auto-aggregation capacity among *L. acidophilus* M92 isolates. In another study, Golowczyc et al. [69] demonstrated a decrease in adhesion to *Saccharomyces lipolytica* cells for an *L. kefir* isolate after removal of the S layer. In addition, several studies have shown that pH, temperature and intergeneric interactions can influence these reactions [64].

### Determination of the cell surface hydrophobicity of pathogenic microorganisms after incubation with the biosurfactants produced by *L. jensenii* P_6A_ and *L. gasseri* P_65_

To examine changes in the cell surface that occurred after the pathogens were exposed to the biosurfactants, the cellular hydrophobicity of the cells was quantified. The percentages of *C. krusei, S. saprophyticus* and *E. aerogenes* cells that adhered to hexadecane were 47, 11 and 2%, respectively, after 24 h of incubation with the biosurfactant produced by *L. jensenii* P_6A_ (data not shown). For the biosurfactant produced by the *L. gasseri* P_65_, the percentages for *E. coli* and *E. aerogenes* were 47 and 1.5%, respectively. These data indicate that the biosurfactants altered the cell surface of these pathogens by changing their adhesion to the hydrophobic substrate. For other microorganisms, the percentage of adherence to hexadecane was comparable to the controls. Previously, it was suggested that the hydrophobicity of the microbial cell, including viral particles can drive the adhesion of many pathogens [70].

## Conclusions

In this study, biosurfactants produced by two *Lactobacillus* strains, *L. jensenii* P_6A_ and *L. gasseri* P_65_, were characterized. The emulsifying activities of biosurfactants from these strains were stable at different pH values (2 at 10) and high temperatures (100 °C). However, emulsifying activity was destabilized in the presence of NaCl, which was not observed employing various concentrations of KCl and NaHCO_3_. Although the auto-aggregation and co-aggregation data obtained for these *Lactobacillus* strains with pathogens may not prove to be very effective in protecting the vaginal mucosa, *L. jensenii* P_6A_ and *L. gasseri* P_65_ produced biosurfactants with considerable antimicrobial activities against *E. coli* and *C. albicans* and anti-adhesive activity against *E. coli, S. saprophyticus* and *E. aerogenes*. These results indicate the potential use of these biosurfactants as alternative antimicrobial agents in medicine for applications against pathogenic microorganisms that are responsible for infections and diseases in the gastrointestinal and urogenital tracts and the skin.
